# Toward an Evidence-Based Definition and Classification of Carbohydrate Food Quality: An Expert Panel Report

**DOI:** 10.3390/nu13082667

**Published:** 2021-07-31

**Authors:** Kevin B. Comerford, Yanni Papanikolaou, Julie Miller Jones, Judith Rodriguez, Joanne Slavin, Siddhartha Angadi, Adam Drewnowski

**Affiliations:** 1 OMNI Nutrition Science, Davis, CA 95616, USA; 2 Nutritional Strategies Inc., Nutrition Research & Regulatory Affairs, Paris, ON N3L0A3, Canada; papanikolaou.yanni@gmail.com; 3 Emerita, Department of Nutrition and Exercise Science, St. Catherine University, St. Paul, MN 55105, USA; jmjones@stkate.edu; 4 Department of Nutrition & Dietetics, Brooks College of Health, University of North Florida, Jacksonville, FL 32224, USA; jrodrigu@comcast.net; 5 Department of Food Science and Nutrition, University of Minnesota, St. Paul, MN 55108, USA; jslavin@umn.edu; 6 School of Education and Human Development, University of Virginia, Charlottesville, VA 22904, USA; ssa2w@virginia.edu; 7 Center for Public Health Nutrition, University of Washington, Seattle, WA 98195, USA; adrewnow@fredhutch.org

**Keywords:** carbohydrate quality, carbohydrate foods, nutrient profiling, fiber, whole grain, glycemic index, glycemic load, diet quality, nutrient density, food groups

## Abstract

Carbohydrate-containing crops provide the bulk of dietary energy worldwide. In addition to their various carbohydrate forms (sugars, starches, fibers) and ratios, these foods may also contain varying amounts and combinations of proteins, fats, vitamins, minerals, phytochemicals, prebiotics, and anti-nutritional factors that may impact diet quality and health. Currently, there is no standardized or unified way to assess the quality of carbohydrate foods for the overall purpose of improving diet quality and health outcomes, creating an urgent need for the development of metrics and tools to better define and classify high-quality carbohydrate foods. The present report is based on a series of expert panel meetings and a scoping review of the literature focused on carbohydrate quality indicators and metrics produced over the last 10 years. The report outlines various approaches to assessing food quality, and proposes next steps and principles for developing improved metrics for assessing carbohydrate food quality. The expert panel concluded that a composite metric based on nutrient profiling methods featuring inputs such as carbohydrate–fiber–sugar ratios, micronutrients, and/or food group classification could provide useful and informative measures for guiding researchers, policymakers, industry, and consumers towards a better understanding of carbohydrate food quality and overall healthier diets. The identification of higher quality carbohydrate foods could improve evidence-based public health policies and programming—such as the 2025–2030 Dietary Guidelines for Americans.

## 1. Introduction

Three crops; rice, wheat, and maize provide about 40% of dietary energy in the global food supply [1]. Despite their importance to the global diet, the impact of carbohydrates on human health has been difficult to determine [2,3,4]. Nutrient-rich carbohydrate foods (CF), including fruits, vegetables, legumes, whole grains, nuts, seeds, and starchy roots and tubers—form the foundation of healthy eating patterns throughout the world [5]. On the other hand, sugar-containing sodas, desserts, and candies—are typically regarded as nutrient poor and their consumption is discouraged [6]. Studies with diets either high or low in carbohydrate content give varying results with respect to body weight, disease risk, and mortality [2,3,4], suggesting that it is CF type and quality that count most for health and longevity [7]. Currently, there is no standardized or unified way to assess the quality of CFs for the overall purpose of improving diet quality and health outcomes, creating an urgent need for the development of metrics and tools to better define and classify high-quality carbohydrate foods.

The primary objectives of this report are to: (1) examine current criteria used to determine macronutrient quality, (2) review evidence that links CF quality to nutrient intake, diet quality, and health outcomes, and (3) propose next steps for developing quantitative metrics to assess CF quality. The present manuscript was informed by (virtual) discussions among members of the Quality Carbohydrate Coalition—Scientific Advisory Council (QCC-SAC), a group with expertise in carbohydrate research, epidemiology, nutrient profiling, and cultural competency. Building on related 2019 work by the Alliance for Potato Research and Education (APRE) [8], QCC-SAC members were interested in advancing scientific understanding of the unique and diverse roles that CFs play in diet quality and healthful eating patterns.

## 2. Carbohydrate Foods: More Than Just Simple or Complex Carbohydrates

CFs are extremely heterogeneous, covering a wide range of foods which contain different types and combinations of carbohydrates [8]. These carbohydrates can be classified in terms of their basic chemical structure (simple vs. complex), their fiber content (e.g., high-fiber vs. low-fiber; soluble vs. insoluble fiber; cereal vs. vegetable fiber), their level of intactness (whole vs. refined) and structure in their food matrix, such as whether they occur intrinsically within a food or not (e.g., naturally occurring vs. added sugars and fibers), their impact on blood glucose and energy metabolism, and their bioactive and functional properties (e.g., prebiotics, resistant starches) (Table 1).

In addition to their carbohydrate content, CFs may also contain varying types and combinations of proteins, fats, vitamins, minerals, phytochemicals, probiotics, and anti-nutrients that can be measured and may impact health. However, only a few of these attributes are generally mentioned in dietary guidelines or reflected in approved food labels. The U.S. Nutrition Facts panel dedicates up to five lines to aspects of carbohydrate. Labels include the amount of total carbohydrate, as well as fiber content, total sugars, added sugars, and sugar alcohols [9]. The Food and Drug Administration (FDA) has also approved several health claims related to carbohydrates that are based on the presence of fruits, vegetables, whole grains, and/or fiber [10]. However, it is not clear whether these health claims are associated with improvements in dietary choices or higher CF quality intake, or even if the foods carrying these claims are beneficial for health. After more than a decade of negative media attention, many consumers only associate carbohydrate-rich foods with the presence of unhealthy ingredients and adverse health effects [11]. Given the complexity and confusion regarding carbohydrate intake and health, a more evidence-based approach to defining and classifying carbohydrate quality could prove useful in helping to inform national food and nutrition policy, with the ultimate goal of guiding individual consumers towards healthier food choices.

## 3. Examination of Current Criteria Used to Define Macronutrient Quality

A basic question that the QCC-SAC aimed to address in their discussions was whether the development of a carbohydrate food quality metric should attempt to follow a protein quality index (PDCAAS) example or make distinctions analogous to those between “healthy” and “unhealthy” fats. For fats and proteins, a main distinction used to determine quality is food source (i.e., distinguishing between plant- and animal-sourced foods). By contrast, carbohydrates are found primarily in plants, with few exceptions besides the sugars in mammalian milks and in honey. Therefore, the designation of CFs by food group (e.g., fruits, vegetables, grains, legumes) rather than source (i.e., plant vs. animal) could prove a useful starting place for assessing CF quality since each food group tends to provide a unique set of nutritional contributions to diet and health [12,13]. Several other factors were also considered by the QCC-SAC when comparing between macronutrient quality assessments (Table 1), with only a few of those factors from protein and fat quality measures (e.g., nutrient content, nutrient ratios, food form, degree of processing) being applicable or potentially useful for assessing CF quality. 

### 3.1. Nutrient-, Food-, and Health-Based Indicators—Lessons from Protein Quality Scores

Nutrient-based, food-based, and health-based measures can all provide valuable information related to food and diet quality, but they do not provide the same information nor are they always consistent with each other. As mentioned previously, while nutrients are the major component of foods, they are not the only aspects that dictate food quality or health effects. Using protein quality scores as an example, the protein digestibility corrected amino acid score (PDCAAS), designates most animal-based proteins as high-quality and most plant-based proteins as lower-quality. However, the value of a protein source cannot be completely captured by its amino acid profile and digestibility, as non-protein factors such as the composition of micronutrients, bioavailability of antioxidants, and overall structure and intactness of the food matrix are also major mediating factors of a food’s overall quality. Simply put, “high-quality proteins” and “healthy protein foods,” describe two different concepts—one relates to the specific chemistry and composition of a food or ingredient, while the other denotes how the food or ingredient will interact with an individual’s biochemistry [14]. So, whilst fat- and sodium-rich processed meat products might contain all the essential amino acids in the right combinations to be classified as a source of “high-quality proteins,” the regular consumption of these products is not generally considered to be part of dietary guidance for healthy diets. However, it would be much more ideal from a public health perspective, if the definitions of a “high-quality food” and a “healthy food” were closely aligned (and even interchangeable with each other). Yet, the alignment of these definitions is challenging. When moving from a nutrient- or food-focused assessment of quality to one that includes health outcomes, the focus will shift from the food’s inherent characteristics (e.g., nutrients, food group, bioactive content, physical form) to also include the consumer’s biological and behavioral characteristics (e.g., genetics, glycemic response, consumption patterns, health status, activity levels, underlying gut microbiota) and the role of the food in the overall diet. This shift in focus from nutrient and food qualities to health impacts introduces an exponential number of personalized variables that are not currently well understood, are not available in standardized public food databases, and are not addressed in current national dietary guidance or other public health platforms. While the use of these precision/personalized tools and technologies would be ideal for informing food quality metric development, this topic will need to be revisited in the future once more infrastructure is in place for collecting, analyzing, and sharing this type of information. 

### 3.2. Carbohydrate Food Quality Indicators 

As a thought-exercise the QCC-SAC expanded on a table that was produced from the APRE’s 2019 meeting comparing carbohydrate quality indicators [8]. The QCC-SAC built out the table to include additional focus areas to consider for assessing CF quality, as well as several additional indicators that could potentially be considered for inclusion in the development of a CF quality metric (Table 2). The final list of potential indicators covered more than two dozen options spread out across the 5 different focus areas of: (1) food chemistry and composition, (2) food and meal consumption context, (3) physiological responses, (4) diet quality and health outcomes, (5), and sustainability impacts. The Panel agreed that no single indicator listed could provide adequate detail regarding the overall quality of a CF. Therefore, the Panel determined that multiple measures of the different aspects of carbohydrate quality should be included in a CF quality metric. The Panel also agreed to wait to incorporate sustainability impacts into the CF quality metric until more publicly available data are available in government sanctioned databases such as the Food and Nutrient Database for Dietary Studies (FNDDS).

## 4. Review of the Evidence Linking Carbohydrate Food Quality to Nutrient Intake, Diet Quality, and Health Outcomes

### 4.1. Methodology—Scoping Review of Carbohydrate Quality Indicators

The QCC-SAC conducted a scoping review to explore the scientific literature to understand how carbohydrate quality indicators—as they relate to nutrients, foods, or diets—have been defined and/or applied in the last decade. The PubMed database was searched for publications between April 2011 and April 2021. The following search strategy was applied to terms listed within the titles, abstracts, and keywords of articles: 

(Carbohydrate quality) AND (indicator OR metric OR index OR score). 

We only included studies published in peer-reviewed journals in English. All study designs were eligible for inclusion except for conference abstracts, letters to the editor, and editorials. These results were supplemented by articles found by citation searching of relevant articles. The final search results were exported into Endnote^®^ reference management software, and duplicates were removed. Studies were excluded if they did not provide a clear definition or measure for assessing carbohydrate quality. 

### 4.2. Results from Scoping Review

**Figure 1:**
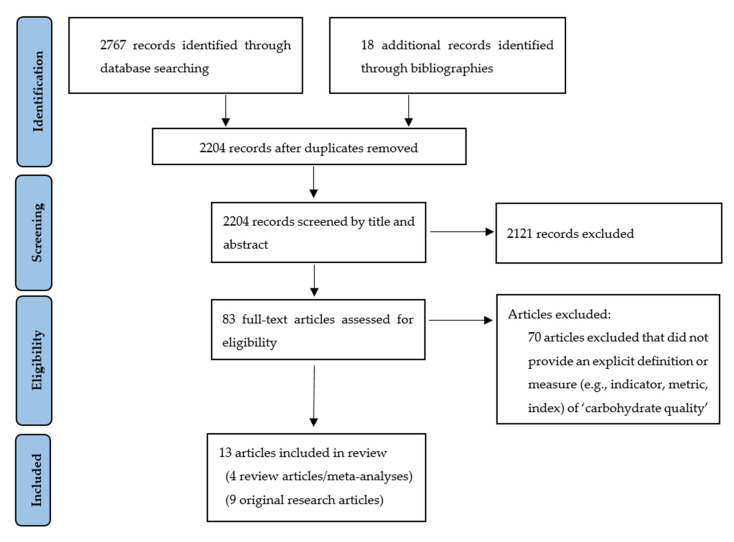
Flow Diagram Describing Article Selection Process.

Thirteen articles were included in the scoping review [15,16,17,18,19,20,21,22,23,24,25,26,27,28] (Figure 1). Five of the 13 articles used the same carbohydrate quality index for assessing different health outcomes [15,16,17,19,20,21]. Only one of these five articles is listed in Table 3 below to avoid redundancy [19]. Therefore, Table 3 contains nine articles, each showing a novel combination of carbohydrate quality definitions (e.g., indicators, metrics, indices, scores) used and type of outcome variable assessed (i.e., nutrient-based, food-based, health-based). The articles in Table 3 showed considerable range in their parameters (e.g., based solely on the carbohydrate content of foods; focused only on GI; inclusive of food form; and/or based on various combinations of nutrient, food, and health indicators). A minority of these articles were focused on nutritional outcomes [18,28], while the majority focused on physiological and health outcomes such as glycemic effects [22], inflammatory markers [23], waist circumference [26], and cardiometabolic disease [24,27].

The 2020 study by Liu et al., tested the ability of different carbohydrate ratios to assess CF quality in more than 2200 processed carbohydrate-rich foods listed in the 2013–2016 National Health and Nutrition Examination Survey (NHANES)/Food and Nutrient Database for Dietary Studies (FNDDS) [28]. This study found that a 10:1 carbohydrate:fiber ratio was one of the simplest and most effective ratios tested for establishing carbohydrate quality (as defined by nutrient contributions) and could be used to identify processed CFs with higher nutritional quality. However, a problem with using the 10:1 carbohydrate:fiber ratio is that when applied to non-processed CFs it could eliminate potentially high-quality CFs (e.g., brown rice and potatoes), which may not meet the fiber criteria (1 g of fiber per 10 g of carbohydrate) but are nonetheless rich in various micronutrients and bioactive compounds that may benefit human health [29,30]. Additionally, the 10:1 carbohydrate:fiber ratio does not distinguish between sugar and starch present in a CF, which may be of critical importance for health. However, other metrics tested in Liu’s study do allow for this differentiation for free sugars (e.g., a 10:1:1 carbohydrate:fiber:free sugar ratio) and do not show exceptionally different results from the simpler 10:1 ratio. The authors also suggest that the use of a simple 10:1 carbohydrate:fiber ratio could help identify processed CFs with higher amounts of protein and minerals, as well as those with lower fat, free sugars, sodium, and calories—therefore providing valuable nutrient profiling information that goes beyond carbohydrate content and captures several nutrients of concern for under- and over-consumption. This type of simple and easy-to-use ratio may therefore have value as a key component for inclusion in metrics for assessing overall CF quality, and not just for the carbohydrate-rich processed products that were tested. 

Several research studies dating back to 2014 have used a multidimensional tool known as the Carbohydrate Quality Index (CQI) to assess the quality of carbohydrates in a dietary pattern [15,16,17,18,19,20,21]. The CQI is based on four carbohydrate quality indicators: (1) total dietary fiber intake, (2) glycemic index (GI), (3) whole grains:total grains ratio, (4) solid carbohydrate:total carbohydrate ratio. Zazpe et al. originally applied this tool to assess micronutrient intake adequacy in 16,841 participants from the Seguimiento University of Navarra (SUN) prospective cohort study [18]. The results from this study indicated that consumers with the highest CQI scores, meaning that their diets were higher in fiber and whole grains while being lower in liquid and high-glycemic carbohydrates, had significantly higher micronutrient intake than those who consumed diets with the lowest CQI scores. Several additional evaluations of CQI in the context of health outcomes have also been conducted in the last 5 years on participants from the SUN study and show promise for the index’s efficacy as a tool for dietary guidance [15,16,17,19,20]. For example, higher CQI diets have been associated with lower risk of general obesity [17,20], abdominal obesity [20], cardiovascular disease incidence [19], depression [16], and all-cause mortality [15], when compared to lower CQI diets. These findings suggests that higher CQI scores are associated with better physical and mental health outcomes, however, the body of research also revealed that the individual components of the CQI may not always be associated with these same benefits. Notably, in an analysis of 19,083 middle-aged participants from the SUN study, when each individual component of the CQI was investigated in relation to mortality, there were no significant differences in mortality between the highest and lowest scores for total dietary fiber intake, GI, whole grains:total grains ratio, or solid carbohydrate:total carbohydrate ratio after 12.2 years of follow-up [15]. It was only when dietary carbohydrate quality was assessed by CQI in its totality that lower mortality risk became apparent in the highest versus lowest quality groups. Although more research is needed, these results suggest that a composite scoring system that considers multiple aspects of carbohydrate quality may uncover dietary impacts that are not revealed by individual indicators. A potential next step in developing a comprehensive CF quality metric could be to convert the CQI, or multiple parts of the CQI, from a diet-focused index to one that can assess the quality of individual CFs.

In a 2021 study, both the 10:1 carbohydrate:fiber ratio and the CQI were compared, along with other indicators of carbohydrate quality by Sawicki et al. to determine their relationships with long-term changes in waist circumference [26]. The study examined the diets of 3101 participants in the Framingham Offspring cohort and found that after 18 years of follow-up, participants who were in the highest quartiles for total fiber intake, CQI score, and low carbohydrate:fiber ratio, all had smaller increases in waist circumference compared to participants in the lowest quartiles. After nearly two decades of dietary analysis, the researchers concluded that both CQI and carbohydrate:fiber ratios could be useful measures for assessing carbohydrate quality, but the simpler 10:1 carbohydrate:fiber ratio is a more useful measure as it could be more easily understood by the general public.

Several other research groups have identified key carbohydrate quality indicators in their work, with most publications including total dietary fiber, carbohydrate:fiber ratio, GI and/or glycemic load (GL) as important elements [23,24,25] (Table 3). In 2015, an international panel of nutrition experts known as the International Carbohydrate Quality Consortium (ICQC) produced a consensus report defining carbohydrate quality in terms of GI/GL with limited focus on other quality factors [22]. This group suggested that postprandial glycemia, which can be predicted from GI/GL, is the most important factor determining the effects of CFs on human health. In more recent publications, the ICQC has also emphasized that fiber amount, fiber type, food source, degree of processing, solubility, and associated compounds, (e.g., micronutrients and bioactive food components) may all influence a CFs’ effects on health [31], in manners both dependent and independent of its effects on GI/GL. Based on the totality of research on carbohydrate quality metrics reviewed above, a deeper dive into the advantages and disadvantages of the most commonly used indicators (GI/GL, total fiber, carbohydrate:fiber ratios) in CF quality metrics is warranted.

### 4.3. Glycemic Index/Load Considerations

Most of the publications reviewed above consider GI and/or GL to be important indicators for assessing CF quality as both measures aim to predict a person’s glycemic response to a food [15,16,17,18,19,20,21,22,23,24,25,26]. However, GL will provide a better picture of how a food will affect blood sugar as it is determined from using the GI value and the amount of carbohydrate present. But context is critical in this regard, with both GI and GL scores varying considerably depending on the biological and behavioral characteristics of the person or population being assessed [8]. GI was designed for a specific research purpose; to rank the glycemic effects of different foods in comparison to pure glucose. The index was then co-opted to guide food selection in patients with Type 2 Diabetes (T2D), and is now in mainstream use for all kinds of health and dietary purposes, despite the fact that GI appears to be a fairly unreliable indicator for the general population. An investigation into GI by Zeevi et al., which intended to characterize the variability of postprandial glycemic responses (PPGR) in 800 individuals, reported that the GI of bread normalized to glucose, ranged up to five-fold between the bottom and top 10% of participants [32], clearly showing that GI depends heavily on the individual consumer’s characteristics (e.g., age, weight, physical activity, genotype, the gut microbiome). Wolever and colleagues demonstrated several insufficiencies in the reliability of the GI in healthy subjects in a study which assigned seven different labs to test the GI of five centrally provided foods [33]. The range of GIs that were derived during this investigation varied by 1.5- to 2-fold between research labs. For example, the GI of rice ranged from a low of 55 to a high of 87, and the GI of barley ranged from 23 to 47, suggesting that using the same protocol with different subjects may lead to major differences in GI values. A recent systematic review of international GI/GL tables published in the American Journal of Clinical Nutrition showed an even greater variation for rice (GI range: 19–116). This 6-fold difference in the GI of a single category of CFs further highlights the unreliability of GI as a food or diet quality assessment tool [34]. The same publication also found wide variations in the GI of breads, breakfast cereals, and carbonated beverages, with approximately 1/3rd of these food categories having a low GI, 1/3rd having a moderate GI, and 1/3rd having a high-GI. While GI testing protocols have become more standardized over time, these large variations in GI values are still difficult to control and could be due to several factors, including regional variations in manufacturing processes, cooking methods, and an ever-growing diversity of products (some of which are not representative of the category) [34]. Variation can also arise when consumer characteristics are considered. Taken together, these different types of study designs reveal that wide variations in GI values depend on both the food’s properties and the consumer’s characteristics, making GI a less than ideal indicator of CF quality, and one that would likely become even less ideal when assessed in the presence of additional variables such as during more complex eating situations (i.e., mixed meals). One such study, which tested the effects of adding 50 g of either carbohydrate, protein, fat, or fiber to the standard 50 g white bread challenge, found that both the GI/GL of CFs were drastically changed in completely opposite directions in response to additional carbohydrates and protein; with additional carbohydrates significantly increasing GI and GL (*p* = 0.0066 and *p* < 0.0001, respectively) and protein significantly reducing them (*p* = 0.0139 and *p* = 0.0140, respectively) [35]. 

While GI may not be the most reliable measure for assessing CF quality, there is no doubt that an individual’s glycemic response to CF intake can be an important factor to consider for metabolic health. Two recent systematic reviews show that lower GI/GL diets are associated with better health markers in individuals with, or at risk for T2D [36,37], however, the evidence is much less clear for other disease states and for healthy populations [25]. In fact, the health effects of low GI/GL diets appear highly variable, with a recent systematic review of 73 studies showing no discernable effects of GI on health outcomes [38]. Furthermore, a recent series of systematic reviews and meta-analyses focused on carbohydrate quality and health outcomes rated the evidence as “low to very low” regarding the long-term effects of GI/GL on health outcomes such CVD, T2D, colorectal and breast cancer [25]. The same publication rated the overall evidence linking whole grain content to health as “low to moderate” and total fiber as “moderate to high”. 

### 4.4. Total Fiber and Carbohydrate:Fiber Ratios

Whether expressed as an absolute value or as a ratio to other nutrients, fiber-specific indicators of carbohydrate intake are the most commonly used measures of carbohydrate quality found in the scientific literature (Table 3). Total fiber intake [18,19,23,24,25,26], carbohydrate:fiber ratio [26,28], total cereal fiber [24,27], and carbohydrate:cereal fiber ratio [24] have all proven to be useful indicators of carbohydrate quality that track with nutritional intakes and health outcomes. Of these indicators, total fiber intake, is the most common and consistent measure of CF quality in the literature. Total fiber is also one of the most inclusive measures, as more specific indicators focused on cereal fiber or cereal fiber ratios will not be applicable to CFs such as fruits, vegetables, legumes, nuts, and seeds. 

In the U.S., total fiber content is required on government-mandated Nutrition Facts panel and can also be found in national food and nutrient databases. Total fiber may also be an ideal indicator for use in a CF quality metric as it is the most robust fiber-related indicator and is already emphasized for improving diet quality in the most recent Dietary Guidelines for Americans 2020–2025 (DGA) [6] and its corresponding Healthy Eating Index (HEI). All of these factors make total fiber one of the most useful and easy-to-use indicators for assessing CF quality. The QCC-SAC acknowledges the contributions of specific fiber types (e.g., cereal fiber, soluble/insoluble fiber, intrinsic/added fibers, fermentable fibers, resistant starches) and their ratios towards various health outcomes, and would aim to include this granular level of information in future iterations of a CF quality metric as greater evidence becomes available.

## 5. Next Steps for Developing Quantitative Metrics to Assess Carbohydrate Food Quality

Based on the literature reviewed and their panel discussions, the QCC-SAC came to several conclusions on how to proceed with developing a standardized metric for characterizing CF quality. The group determined that the best approach would be to select and harmonize multiple indicators that focus strictly on the intrinsic qualities (i.e., nutrient-based and food-based indicators) of CFs, and not on their dynamic and difficult to predict interactions with human biology and behavior. While a metric based on the intrinsic qualities of CFs has limitations and cannot fully capture the value of these foods towards physiological responses or health outcomes, this type of assessment can more feasibly be developed and depended on than one which is based on extrinsic factors. The group agreed that the intrinsic indicators of most interest for assessing CF quality were: (1) total fiber content, (2) nutrient density, (3) nutrients of public health concern for overconsumption and underconsumption according to the 2020–2025 DGA [6], (4) food group designation, (5) food matrix effects, (6) processing effects, and (7) and bioactive food components. In further panel discussions, this list was narrowed down to three components to consider for inclusion in the initial metric based on ease of use, feasibility, and availability of standardized data from sources such as the USDA’s FoodData Central. These three components were: (1) total fiber, (2) nutrient density with an emphasis on nutrients of public health concern, and (3) food group designation. All three of these components can be addressed through nutrient profiling (NP) techniques.

### Nutrient Profiling—A Method to Assess the Nutrient Density of Foods

NP methods are used to rate or rank individual foods according to their energy and nutrient content. Foods deemed to be nutrient-dense or nutrient-rich are those that contain relatively more nutrients to encourage relative to calories. In the last few decades, NP models such as Food Standards Agency (FSA) Ofcom, Nutri-Score, Food Standards Australia New Zealand (FSANZ), WHO Regional Office for Europe (EURO), and WHO Regional Office for the Americas/Pan American Health Organization (PAHO) have provided the scientific basis for a wide variety of educational, regulatory, and taxation activities [39]. NP models have become benchmarks for the implementation of dietary guidance, development of nutrient-focused warning labels, adjudication of nutrition and health claims, and determination of foods that can be advertised and marketed to children [40]. 

Ultimately, the goal of NP models is to assist in the implementation of dietary guidance by helping to identify foods that are healthy, affordable, accessible, and nutrient-rich. The development of these models requires access to reliable databases focused on the nutrient composition of foods. When developing a CF quality metric, the foundational components ought to aim align with both the FDA regulations (which are nutrient-based) and the current DGA guidance (which aims for a food-based, dietary patterns approach), as this alignment will likely improve the metric’s translatability and ease of use for stakeholders (researchers, educators, industry, policymakers) and consumers.

## 6. Main Principles for Carbohydrate Food Quality Assessment and Metric Development

(1) Overall, the QCC-SAC recognized seven principles that could help guide the development of a standardized metric for characterizing CF quality: Assessment of CF quality should be based on nutrient composition data that are accurate, reliable and in the public domain. The USDA FoodData Central is a valuable resource that fits these criteria.
(2) CFs come from many sources, most of which are plant-based. A nutrient profiling model that is both food group and category specific is ideal.
(3) CF quality can be driven by multiple factors from enrichment and fortification, to added sugar content and sodium: potassium ratios. These factors should be further investigated for inclusion in a CF quality metric.
(4) CF quality assessment should be based on nutrient- and food-based indicators until more reliable data are available.
(5) The CF quality metric should utilize a holistic approach, incorporating new data on topics such as affordability, bioactive compound content, and environmental impacts as they become available. Much more data and evidence will be required before most of these are additions are possible.
(6) The CF quality metric should be aligned with, and be able to operate within, already validated dietary guidance tools such as food-based dietary guidelines (FBDGs), FDA-approved nutrition and health claims, and on-package food labels. The FDA does not view GI/GL as a characteristic of the food alone, nor does it have a database dedicated to GI or GL values.
(7) The CF quality metric should be easily expandable and able to accommodate additional inputs as new evidence becomes available.
(8) The CF quality metric should be culturally inclusive, translatable, and easy-to-use. The main goal of the metric is to help with the implementation of dietary guidelines in promoting better food choice for improved diet quality and health.

## 7. Conclusions

Despite the array of indicators available for assessing CF quality, there is currently no gold-standard or validated method for doing so. In essence, there are dozens of carbohydrate characteristics to be considered when assessing overall CF quality, but there are only a few that can easily be measured, standardized, or presently applied to CFs. Some of the most common indicators guiding consumer food choices are total fiber, whole grain content, and GI/GL, yet each of these indicators have their limitations and are only able to provide a piece of a much bigger picture. For example, total fiber does not capture the differences in functions between soluble and insoluble forms; measures of whole grain content can only be applied to a single food group (i.e., grains); and GI/GL can vary significantly depending on the biology and behavior of the consumer. A more promising approach for informing public policy, public health professionals, and consumers on quality CF intake would be to develop a composite measure, aimed at harmonizing information from multiple CF quality indicators into a single easy-to-use tool that can help identify higher quality CFs.

Approaching CF quality from the perspective of nutrition scientists and physiologists, the QCC-SAC fully appreciates the intentions of this work to provide tools that may inform healthier food choices and improve human health, ideally and eventually within a healthy, sustainable diets framework. However, after assessing the current body of scientific evidence, the QCC-SAC concluded that the most pragmatic approach to this project would be to first establish a multi-component metric based exclusively on the intrinsic qualities of CFs (i.e., selected nutrient-based and food-based indicators), and only once that is completed, to then attempt to expand the metric to characterize CFs in terms of their potential effects on human health outcomes and sustainability impacts. Future QCC-SAC manuscripts are planned to address metric development and applications. Once the initial metric is set, the goal is for it to be tested and revised as necessary by experts until a universally accepted version can be validated and integrated into dietary guidance tools.

## Figures and Tables

**Table 1:** Comparison of Macronutrient Quality Indicators.

	Protein	Fat	Carbohydrates
Common Descriptive Indicators for Determining Macronutrient or Food Quality	Plant/Animal-Sourced Complete/Incomplete Essential Amino Acids	Plant/Animal-Sourced	Food Groups (e.g., Grains, Vegetables, Legumes, Fruits, Discretionary Foods)
	High/Low in Branched-Chain Amino Acids	Saturated/MUFA + PUFA	Whole Grain/Refined Grain/ Enriched Grain
	The Protein Digestibility-Corrected Amino Acid Score (PDCAAS)	Solid/Liquid at Room Temp	Simple/Complex
	Lean/Fatty Protein Foods	Short-, Medium-, Long-Chain Fatty Acids	High/Low in Fiber
		Trans/Cis Bonds	Processed/Unprocessed
		Omega-3 or Omega 3:6 Ratio	Solid/Liquid Form
			Intrinsic/Added Sugars
			Intrinsic/Added Fiber
Common Physiological Measures and Health Outcomes for Determining Macronutrient or Food Quality	Protein Synthesis Rates	Postprandial Lipemia	Glycemic Index/Load
	Muscle Mass/Function	Fasting Blood Lipids	Fasting Glucose/HbA1c
	Growth Rate in Children	Insulin Secretion/Resistance	Insulin Secretion/Resistance
	Protein/Calorie Malnutrition	Inflammatory Markers	Hydrogen/Methane in Breath
	Stunting/Wasting	Body Weight	Body Weight
		Type 2 Diabetes	Type 2 Diabetes
		Cardiovascular Disease	Cardiovascular Disease
			Dental Caries
			Gut Microbiota

**Table 2:** Potential Indicators for Assessing the Carbohydrate Food Quality.

Food Chemistry and Composition	Food and Meal Consumption Context	Physiological Responses	Diet Quality and Health Outcomes	Sustainability Impacts
Macronutrient content (types and ratios)	Interactions with other foods and ingredients in a meal or dietary pattern	Glucoregulation (glycemic index/load, insulin response, etc.)	Nutrient adequacy	Public health effects
Food matrix effects	Consumer characteristics (age, health status, other demographic considerations)	Blood lipids (postprandial lipemia, fasting triglycerides, cholesterol, etc.)	Alignment with Healthy Eating Index (HEI)	Environmental impacts
Carbohydrate characteristics (fiber, starch, sugar—types, amounts, and ratios)	Lifestyle factors (level of physical activity, stress, sleep, etc.)	Inflammatory and immune markers	Food group variety or diversity	Socio-Cultural impacts
Micronutrients (especially of public health concern)	Consumption patterns (frequency of intake, speed of ingestion, etc.)	Multi-omics (genomics, proteomics, metabolomics)	Chronic disease risk or incidence	Economic impacts
Bioactive food components (phytonutrients, prebiotics, probiotics)	Food form, processing, and preparation	Gut microbiome	Mortality statistics	

(This is table was adapted and expanded from Kanter 2019 [8]) (Permission has been obtained).

**Table 3:** Selected Examples of Carbohydrate Quality Indicators and their Combinations.

	Nutrient-Based Indicators					Food-Based Indicators			Health-Based Indicators
	Total Fiber	Cereal Fiber	10:1 Carb:Fiber Ratio	Carb: Cereal Fiber Ratio	Starch: Cereal Fiber Ratio	Whole Grain	Whole Grain: Total Carb Ratio	Solid Carb: Total Carb Ratio	Glycemic Index/ Response
Buyken 2014 [23]	X					X			X
Zazpe 2014 [18]	X						X	X	X
Augustin 2015 [22]									X
Zazpe 2016 [19]	X						X	X	X
AlEssa 2018 [27]		X		X	X				
Reynolds 2019 [25]	X					X			X
Hardy 2020 [24]	X	X							X
Liu 2020 [28]			X						
Sawicki 2021 [26]	X	X	X	X			X	X	X

## Data Availability

Data sharing not applicable.
